# FAM115C could be a novel tumor suppressor associated with prolonged survival in pancreatic cancer patients

**DOI:** 10.7150/jca.38399

**Published:** 2020-02-10

**Authors:** Kiyoshi Saeki, Hideya Onishi, Satoko Koga, Shu Ichimiya, Kazunori Nakayama, Yasuhiro Oyama, Makoto Kawamoto, Kukiko Sakihama, Takeo Yamamoto, Ryota Matsuda, Yoshihiro Miyasaka, Masafumi Nakamura, Yoshinao Oda

**Affiliations:** 1 Department of Anatomical Pathology, Graduate School of Medical Sciences, Kyushu University, Fukuoka, Japan; 2 Department of Cancer Therapy and Research, Graduate School of Medical Sciences, Kyushu University, Fukuoka, Japan; 3 Department of Surgery and Oncology, Graduate School of Medical Sciences, Kyushu University, Fukuoka, Japan

**Keywords:** FAM115C, hypoxia, microenvironment, pancreas cancer, tumor suppression

## Abstract

Hypoxia is a characteristic feature of the tumor microenvironment in pancreatic ductal adenocarcinoma (PDAC). We have recently explored new targeting molecules and pathways in PDAC cells under hypoxic conditions. In this study, we performed a microarray experiment to analyze the genes up-regulated in PDAC cell lines under hypoxia compared to normoxia, and identified human family with sequence similarity 115, member C (FAM115C) as a candidate gene for further study. Our data showed that FAM115C was overexpressed in PDAC cell lines under hypoxia, and FAM115C inhibition promoted PDAC cell migration and invasion *in vitro*. FAM115C inhibition did not affect tumor cell proliferation in PDAC. Immunohistochemically, FAM115C expression was observed ubiquitously in normal pancreas, pancreatic intraepithelial neoplasia (PanIN) and PDAC tissue, and it was located mainly in the nucleus but also in the cytoplasm of cells. In qPCR analysis, high expression of FAM115C was correlated with better prognosis in patients with PDAC. Our findings suggest that FAM115C could be a novel tumor suppressor associated with prolonged survival in patients with PDAC.

## Introduction

Pancreatic ductal adenocarcinoma (PDAC) is one of the most aggressive and lethal malignancies, with a median survival <1 year 1,2. One of the reasons for this poor survival may be that many patients cannot undergo curative surgery because their symptoms appear late in disease progression and metastasis has typically occurred by the time of diagnosis. There are still few effective chemotherapeutic agents for the treatment of pancreatic cancer. More effective therapeutic approaches for PDAC are urgently required.

The tumor microenvironment is a key to understanding the initiation and progression of PDAC, and the tumor microenvironment in PDAC is extremely hypoxic 3-7. Considering this hypoxic microenvironment is important for treating pancreatic cancer. Although it has been reported that a hypoxic microenvironment is associated with a poor prognosis for PDAC because of the contributions of particular signaling pathways or molecules that are activated under hypoxia 4,5,8-10, the exact molecular mechanisms and pathways activated under hypoxic conditions remain largely unknown.

According to the U.S. National Center for Biotechnology Information (NCBI) Basic Local Alignment Search Tool (BLAST), the gene known as human family with sequence similarity 115, member C (FAM115C, also known as TCAF2) is a novel gene of uncertain function. FAM115C has been reported to bind to the transient receptor potential 8 (TRPM8) channel, which promotes its trafficking to the cell surface, and FAM115C has a strong promigratory effect on prostate cancer cells, as evidenced by the strong inhibition of cell migration in the presence of siFAM115C 11. However, the biological significance of FAM115C in PDAC cells remains unclear.

In the present study, as a step in the development of therapeutic strategies for pancreatic cancer and to clarify the nature of the tumor environment in pancreatic cancer, we examined the pathobiological role of FAM115C in PDAC progression under tumor hypoxic conditions.

## Materials and Methods

### Cell culture and reagents

Three human PDAC lines (ASPC-1, SUIT-2, and PANC-1) and human normal cell lines (HPDE and TIG) were maintained in RPMI 1640 medium (Nacalai Tesque, Kyoto, Japan) supplemented with 10% fetal calf serum (FCS; Life Technologies, Grand Island, NY) and antibiotics (100 units/ml of penicillin and 100 μg/ml of streptomycin). To establish normoxic conditions, cells were cultured in 5% CO_2_ and 95% air. To establish hypoxic conditions, cells were cultured in 1% O_2_, 5% CO_2_, and 94% N_2_ in a multigas incubator (Sanyo, Tokyo). PDAC cells cultured under normoxia or hypoxia for 6, 12, 24, and 48 hr were used for the experimental analyses described below.

### DNA microarray

The cRNA was amplified, labeled, and hybridized according to the recommended protocol from Agilent (Santa Clara, CA). Relative hybridization intensities and background hybridization values were calculated using Agilent's Feature Extraction software. We calculated Z-scores and ratios from the normalized signal intensities of each probe for the comparison between ASPC-1 cells cultured under hypoxia for 2 days and ASPC-1 cells cultured under normoxia; then, the same comparison was made for SUIT-2 cells.

### Western blot analysis

Western blotting was performed as described previously 12. Whole cell extraction was performed with M-PER reagents (Pierce Biotechnology, Rockford, IL) according to the manufacturer's instructions. Protein concentrations were determined with the Bio-Rad Protein Assay (Bio-Rad, Hercules, CA), and protein samples (50 μg) were separated by electrophoresis on a sodium dodecyl sulfate (SDS)-polyacrylamide gel and transferred to Protran nitrocellulose membranes (Whatman, Dassel, Germany). The protein-transferred membranes were incubated overnight at 4℃ with primary antibodies for FAM115C (1:500, GTX123264; GeneTex, Irvine, CA). Peroxidase-linked secondary antibodies (Amersham Biosciences, Piscataway, NJ) were subsequently added and the membranes were further incubated for 1 hr at room temperature. The antibodies for α-tubulin (1:1000; Sigma-Aldrich, St. Louis, MO) were used as protein loading controls.

### RNA interference

ON-TARGETplus™ SMARTpool siRNA targeting FAM115C (L-018080), HIF-1α (L-004018), and negative control siRNA (ON-TARGETplus™ Control non-targeting siRNA, D-001810) were purchased from Dharmacon (Lafayette, CO). Cells (0.2 × 10^6^ cells/ well) seeded in six-well plates were transfected with 100 nM siRNA under normoxia using Lipofectamine RNAiMAX reagent (Invitrogen, Carlsbad, CA) according to the manufacturer's instructions. Cells were used for experiments at 2 days after transfection. Additional cells were cultured under normoxia and hypoxia in each experiment.

### Cell migration/invasion assay

Cell migration and invasion assays were performed with (the invasion assay) or without (the migration assay) a Matrigel-coated Transwell insert as described previously 13. Briefly, cells (2 × 10^5^) were treated for 48 hr with FAM115C siRNA, then placed in an upper chamber and incubated for 18 hr. The cells that migrated to the lower side of the filter were fixed and stained with Diff-Quik reagent (Sysmex, Kobe, Japan) and then counted under a light microscope (Nikon Eclipse TE 300, Nikon, Tokyo).

### Cell proliferation assay

All PDAC cell lines were seeded onto 96-well plates at 5,000 cells/well and were cultured under normoxia and hypoxia for 24 and 48 hr. Cell proliferation was assessed by absorbance (Biotrak visible plate reader; Amersham Biosciences) at 492 nm (reference wavelength: 620 nm) using Cell Count Reagent SF (Nacalai Tesque). At 48 hr after the FAM115C small interfering RNA (siRNA) transfection, the cells were reseeded onto 96-well plates and the proliferation rate was measured.

### *In vivo* xenograft tumor model

Six-week-old female BALB/c nude mice were obtained from Charles River Laboratories Japan (Kanagawa, Japan) and acclimatized for 2 weeks. All animal procedures were approved by the Animal Care and Use Committee at Kyushu University (A30-314-0). Cultured SUIT-2 cells transfected with FAM115C-targeting siRNA and non-targeting control siRNA were subcutaneously implanted into bilateral flank regions (5.0 × 10^5^ cells in 50 μl of RPMI medium) of the BALB/c nude mice (n=3 in each treatment group). The tumor sizes were measured 2×/week, and the tumor volume was calculated as follows: A × B^2^ × 0.5, where B is the smaller of the perpendicular diameters. The mice were euthanized when the tumor size reached 2 cm in diameter or if the animal became moribund during the observation period.

### Immunocytochemistry

Cells of each cell line (ASPC-1, SUIT-2, and PANC-1) were plated on coverslips in 12-well plates and cultured for 24 hr. The cells were then washed twice with phosphate-buffered saline (PBS), fixed with 4% paraformaldehyde in PBS for 10 min, and washed three times in PBS. The cells were permeabilized with 0.5% Triton X-100 in PBS for 5 min, and washed three times. A 15-min incubation with the reagent Blocking One-P (Nacalai Tesque) was followed by an overnight incubation using the primary antibody FAM115C (1:200, orb183515; Biorbyt, Cambridge, UK) diluted with 10% Blocking One-P in PBS at 4℃. The coverslips were then washed three times with PBS and incubated for 40 min at room temperature with Alexa 488-labeled donkey anti-mouse (1:1000; Invitrogen) diluted with 10% Blocking One-P in PBS. The wells were washed three times in PBS and then mounted on glass coverslips on a microscope slide with Prolong Diamond Antifade Reagent with DAPI (Thermofisher Scientific, Waltham, MA). Images were visualized using a confocal microscope (A-1; Nikon).

### Patients and case selection

We retrospectively reviewed the cases of 307 patients with pancreatic adenocarcinoma who underwent curative surgical resection at the Department of Surgery and Oncology, Kyushu University Hospital between 2007 and 2015. Among them, we retrieved consecutive 62 cases whose frozen tumor samples were well preserved and stored at the Department of Anatomical Pathology, Kyushu University Hospital. To exclude the metastatic carcinoma from other primary lesions, we thoroughly reviewed the retrospective clinicopathologic database. After each patient's surgical resection, a part of a tumor specimen was frozen within 30 min, and the rest of the tumor was formalin-fixed and paraffin-embedded. The clinicopathological profiles of the patients were obtained by reviewing the medical records and database and are summarized in Table 1. The clinicopathologic findings (age, sex, tumor location, tumor size, lymphatic permeation, vessel invasion, perineural invasion, histologic grade, and UICC TNM stage) were evaluated.

This study was approved by the Ethics Committee of Kyushu University (IRB: 30-448) and conducted according to the Ethical Guidelines for Human Genome/Gene Research enacted by the Japanese Government and Helsinki Declaration.

### Immunohistochemistry

We analyzed the samples of the 37 patients with surgically resected pancreatic adenocarcinoma by immunohistochemistry (IHC). The samples of the BALB/c nude mice in which cultured SUIT-2 cells transfected with FAM115C-targeting siRNA and non-targeting control siRNA were subcutaneously implanted were also analyzed by IHC. The IHC was performed using 4-μm-thick formalin-fixed, paraffin-embedded tissue sections and the primary antibody FAM115C (1:100, orb183515; Biorbyt). Endogenous peroxidase activity was blocked by incubation in methanol containing 0.3% H_2_O_2_ for 30 min. Antigen retrieval was conducted by microwave heating for 20 min with citrate buffer (pH 6.0).

The slides were then incubated with the primary antibody (FAM115C) at 4°C overnight and subsequently incubated with biotin-free horseradish peroxidase enzyme-labeled polymer (EnVision plus System, DAKOCytomation, Glostrup, Denmark) for 40 min at room temperature. The labeled antigens were visualized using 3,3-diaminobenzidine tetrahydrochloride as a chromogen. Counterstaining was performed with hematoxylin. Positive and negative controls were used.

For the evaluation of FAM115C expression, we observed 20 microscopic fields at 100× magnification. We evaluated the proportion and the intensity of the immunoreactive cells over the entire slides by using the Allred score (AS) 14. We divided the immunoreactivity into four grades by the AS as follows: Grade 0, an AS of 0; Grade 1, AS of 2-4; Grade 2, AS of 5 or 6; and Grade 3, AS of 7 or 8 15,16.

### Real-time PCR

Frozen tissues of the 62 patients with surgically resected pancreatic adenocarcinoma were powdered under liquid nitrogen using a Multi-beads shocker (Yasui Kikai, Osaka, Japan). Total RNA was extracted using the miRNeasy Mini kit (Qiagen, Venlo, Netherlands) and reversed-transcribed with ReverTra Ace qPCR RT Master Mix with gDNA Remover (Toyobo, Osaka, Japan) according to the manufacturer's protocol.

Total RNA of the ASPC-1, SUIT-2, and PANC-1 cells was extracted using a High Pure RNA Isolation Kit (Roche, Mannheim, Germany) and quantified by spectrophotometry (Ultraspec2100; Amersham Pharmacia Biotech, Cambridge, UK).

For the real-time PCR, 1 μg of RNA was treated with DNase and reverse transcribed to cDNA with a Quantitect Reverse Transcription Kit (Qiagen, Valencia, CA) according to the manufacturer's protocol. Reactions were run with iQ™ SYBR Green Supermix (Bio-Rad) on a StepOnePlus™ Real-Time PCR System (Applied Biosystems, Carlsbad, CA). Each sample was tested in triplicate to confirm the reproducibility of the results. Each experiment was performed in triplicate, with replicates performed on different days. All the experiments were done in triplicate and repeated 3 times. A cycle threshold (Ct) value of 37 was used as the cutoff value to define undetectable/detectable, and thus specimens with a Ct <37 were used in this study 17,18.

The primer sequences used were as follows: for FAM115C, 5'-ACCACGAGAATGGGAACTTG-3' and 5'-GAGCCTGTGCAGGGATATGT-3'; for β-actin, 5'-TTGCCGACAGGATGCAGAAGGA-3' and 5'-AGGTGGACAGCGAGGCCAGGAT-3'. The amount of each target gene in a given sample was normalized to the level of β-actin.

### Statistical analyses

The data are presented as the mean ± standard deviation (SD). The χ^2^ test was used to analyze the tumorigenicity in mice. Calculations were carried out using JMP 12.0 software (SAS Institute, Cary, NC) or Microsoft Excel software (Microsoft, Redmond, WA). Student's *t*-tests and Fisher's exact tests were used to compare continuous variables between pairs of groups. Survival curves were examined by the Kaplan-Meier method, and significance was examined using the generalized Wilcoxon test and the log-rank test. A p-value <0.05 was considered significant.

### Bioinformatics analysis

The publicly available overall survival dataset (containing the published data for 185 patients) and disease/progression-free survival dataset (containing the published data for 141 patients) were obtained from The Cancer Genome Atlas-Pancreatic Adenocarcinoma (TCGA-PAAD), available through the cBio Cancer Genomics Portal (http://www.cbioportal.org/public-portal).

## Results

### FAM115C expression was increased in the PDAC cell lines under hypoxia compared to normoxia

**Figure 1:**
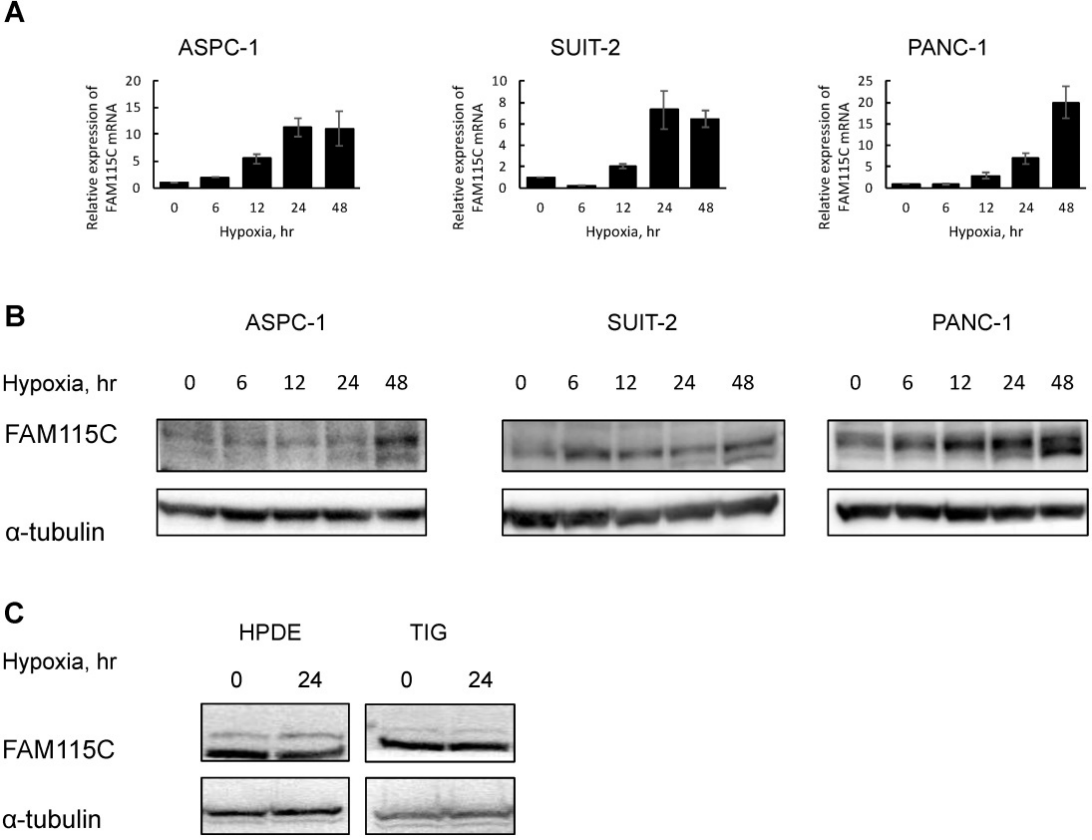
FAM115C mRNA and protein expressions were markedly increased under hypoxia compared to normoxia in each PDAC cell line **(A, B)**. **A:** Real-time PCR analysis of FAM115C mRNA expression in the PDAC lines under hypoxia (1% O2) for 0, 6, 12, 24, and 48 hr. Values in the graph are the mean ±SD. **B:** Western blot analysis of the expression of FAM115C in the PDAC cells under hypoxia (1% O_2_) for 0, 6, 12, 24, and 48 hr. FAM115C protein expressions under hypoxia were similar to those under normoxia in normal cells **(C)**. **C:** Western blot analysis of the expression of FAM115C in the HPDE and TIG cells under hypoxia (1% O_2_) for 24 hr.

We first analyzed the genes up-regulated under hypoxia compared to normoxia in the PDAC cell lines ASPC-1 and SUIT-2 in a microarray experiment. The results revealed that FAM115C was markedly up-regulated under hypoxia compared to normoxia. We selected FAM115C as the candidate gene for further study because the ratio between normoxia and hypoxia was large, and this may have affected the biological functional change of the microenvironment of pancreatic cancer (Table 2). To confirm the microarray analysis finding that FAM115C expression can be induced by hypoxia, we exposed ASPC-1, SUIT-2, and PANC-1 cells to hypoxia for up to 48 hr and then examined the FAM115C expression by real-time PCR and western blotting. The results confirmed that the FAM115C mRNA were markedly increased under hypoxia compared to normoxia in each of the three PDAC lines (Fig. 1A). In addition, the FAM115C protein expressions were markedly increased under hypoxia for up to 48 hr compared to normoxia in ASPC-1 and PANC-1 cells (Fig. 1B). In SUIT-2 cells, the protein expression under each phase of the hypoxic condition (6, 12, 24, and 48 hr) was also higher than that under the normoxic condition (0 hr) (Fig.1B). These results suggested that hypoxia induces FAM115C expression in PDAC cells.

Furthermore, to investigate whether FAM115C expression of normal cells also can be induced by hypoxia, we exposed HPDE and TIG cells to hypoxia for 24 hr and then examined the FAM115C expression by western blotting. In those cell lines, FAM115C expressions by western blotting under hypoxia were similar to those under normoxia (Fig. 1C). The results suggested that up-regulated FAM115C expression under hypoxia compared to normoxia is specific to pancreas cancer cells.

### FAM115C inhibition increased the cell migration and invasion in PDAC *in vitro* under normoxia and hypoxia

**Figure 2:**
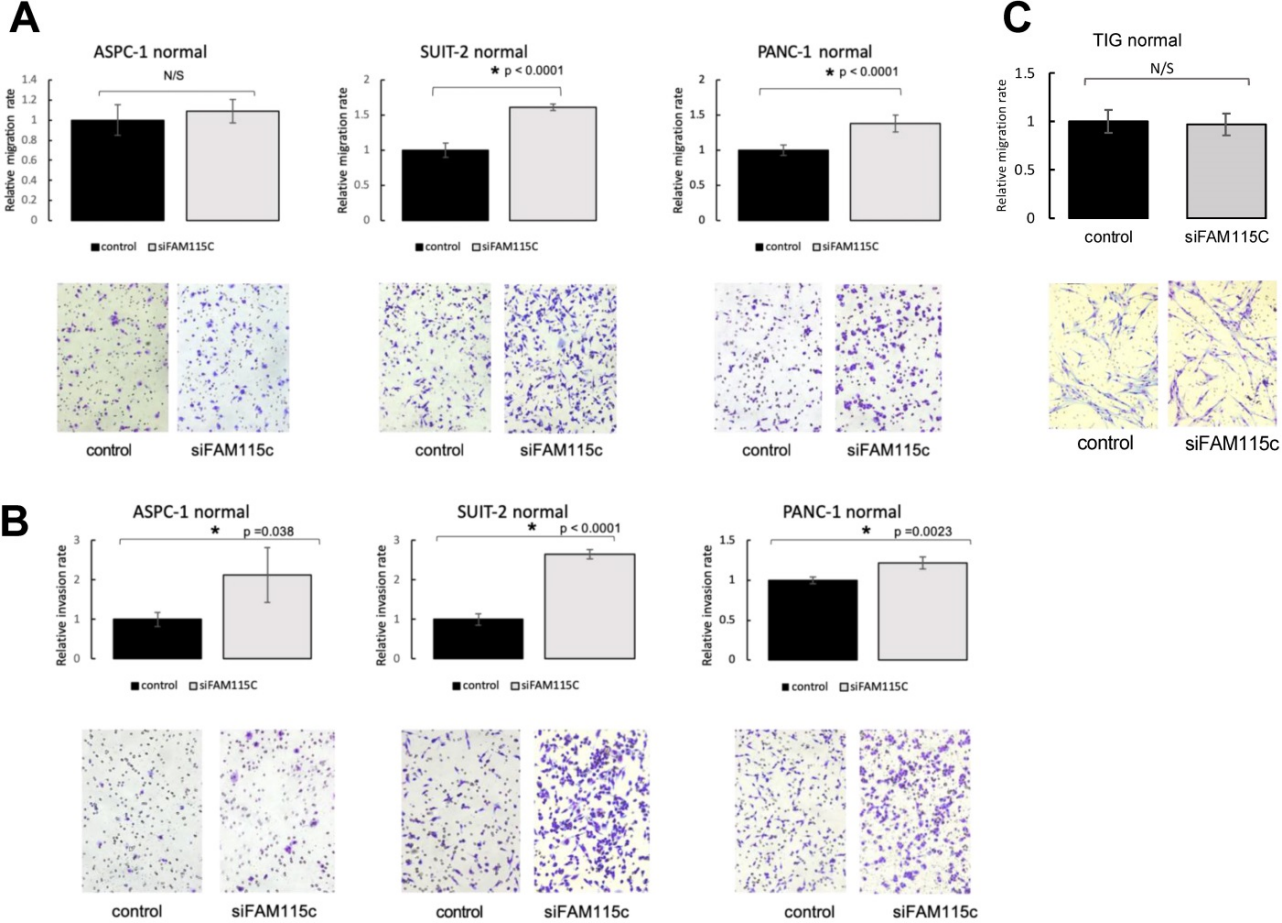
FAM115C is involved with cell migration and invasion in vitro under normoxia. **(A, B)**. **A:** Migration assay of each PDAC cell line transfected for 18 hr with control siRNA or FAM115C siRNA under normoxia. **B:** Invasion assay of each PDAC cell line transfected for 18 hr with control siRNA or FAM115C siRNA under normoxia. FAM115C is not involved with normal cell migration *in vitro* under normoxia **(C)**. **C:** Migration assay of TIG cell line transfected for 18 hr with control siRNA or FAM115C siRNA under normoxia. N/S: not significant. *p<0.05. Bar: SD. Original magnifications x100.

**Figure 3:**
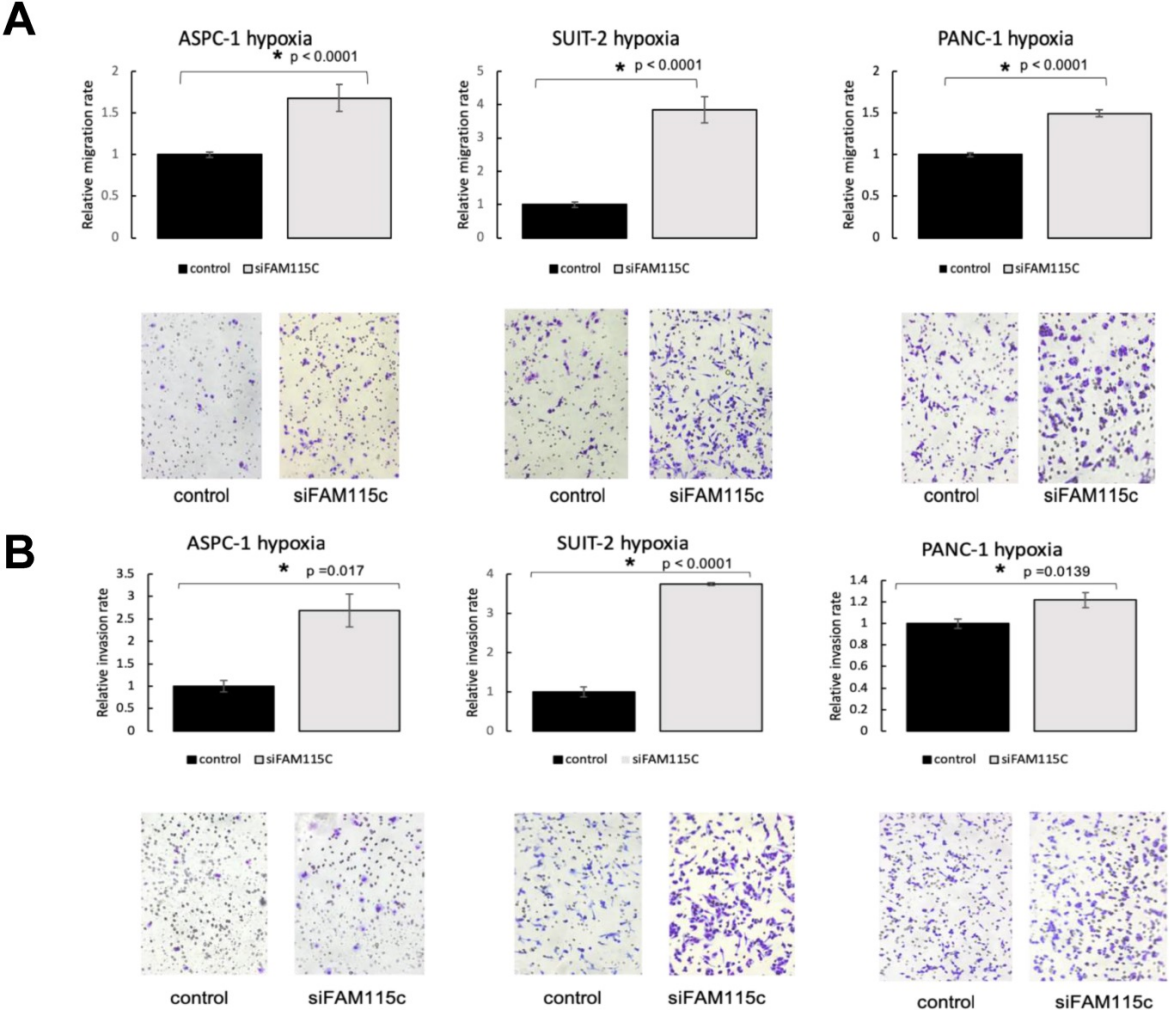
FAM115C is involved with cell migration and invasion in vitro under hypoxia **(A, B)**. **A:** Migration assay of each PDAC cell line transfected for 18 hr with control siRNA or FAM115C siRNA under hypoxia. **B:** Invasion assay of each PDAC cell line transfected for 18 hr with control siRNA or FAM115C siRNA under hypoxia. *p<0.05. Bar: SD. Original magnifications x100.

To determine the effect of FAM115C expression on PDAC migration and invasion, we transfected ASPC-1, SUIT-2, and PANC-1 cells with siFAM115C for 48 hr. The migratory ability of the FAM115C siRNA-transfected SUIT-2 and PANC-1 cells was significantly promoted compared to that of the controls under normoxia (Fig. 2A). The migratory ability of FAM115C siRNA-transfected ASPC-1 cells was not significantly different from that of the control (Fig. 2A). The invasiveness of FAM115C siRNA-transfected ASPC-1, SUIT-2, and PANC-1 cells was significantly promoted compared to that of the controls under normoxia (Fig. 2B). Both the migratory ability and the invasiveness of FAM115C siRNA-transfected ASPC-1, SUIT-2, and PANC-1 cells were also significantly promoted compared to those of the controls under hypoxia (Fig. 3A, B). And, also to determine the effect of FAM115C expression on normal cells migration, we transfected TIG cells with siFAM115C for 48 hr. The migratory ability of FAM115C siRNA-transfected TIG cells was not significantly different from that of the control under normoxia (Fig. 2C). These data implied that FAM115C might act as a tumor suppressor of pancreatic cancer cells with regard to the invasiveness *in vitro*.

### FAM115C inhibition did not affect tumor proliferation in PDAC

**Figure 4:**
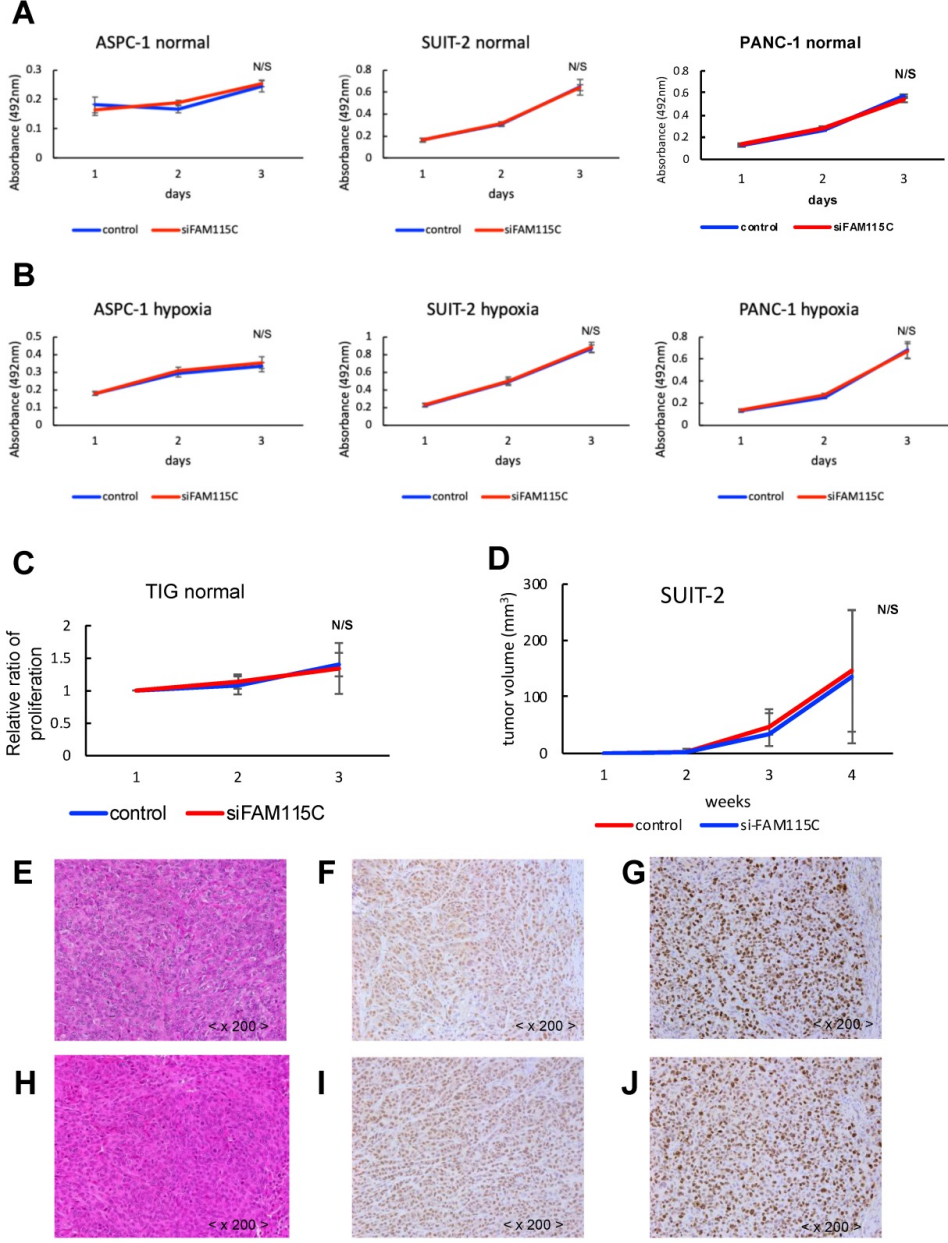
FAM115C does not influence proliferation *in vitro* in PDAC cell lines and normal cells **(A-C)**, or tumorigenicity and tumor proliferation *in vivo* **(D-J)**. **A:** Proliferation assay of each PDAC cell line transfected with control siRNA or FAM115C siRNA under normoxia for 24 and 48 hr. **B:** Proliferation assay of each PDAC cell line transfected with control siRNA or FAM115C siRNA under hypoxia for 24 and 48 hr. **C:** Proliferation assay of TIG cell line transfected with control siRNA or FAM115C siRNA under normoxia for 24 and 48 hr. **D:** The SUIT-2 xenograft tumor volume of the control siRNA group (n=3) and FAM115C siRNA group (n=3). Matrigel mixtures containing 5 × 10^5^ SUIT-2 cells transfected with control siRNA or FAM115C siRNA were implanted subcutaneously in the flank site of BALB/c nude mice (n=3 per group). N/S: not significant. Bar: SD. **E:** Representative image of hematoxylin and eosin (H&E) staining of a SUIT-2 xenograft tumor transfected with control siRNA. **F, G:** Representative immunohistochemical image of FAM115C **(F)** and Ki-67 staining **(G)** of SUIT-2 xenograft tumors transfected with control siRNA. **H:** Representative image of H&E staining of a SUIT-2 xenograft tumor transfected with FAM115C siRNA. **I, J:** Representative immunohistochemical image of FAM115C **(I)** and Ki-67 staining **(J)** of SUIT-2 xenograft tumors transfected with control siRNA. Original magnifications ×200.

We next investigated whether FAM115C affects proliferation in PDAC. The proliferative ability of each of the three FAM115C siRNA-transfected PDAC cell lines was not significantly different from that of the controls under either normoxia or hypoxia (Fig. 4A,B). We also investigated the effect of FAM115C expression on normal cells proliferation. The proliferative ability of FAM115C siRNA-transfected TIG cell line was not significantly different from that of the control under normoxia (Fig. 4C). To assess the impact of FAM115C on tumor growth *in vivo*, SUIT-2 cells transfected with control siRNA or FAM115C siRNA were subcutaneously implanted into bilateral flank regions of athymic nude mice (3 mice per group). There were no significant differences in tumor volume between the control group and the FAM115C-inhibited group (Fig. 4D).

The histopathological findings of the FAM115C-inhibited group were similar to those of the control group (Fig. 4E, H). The immunohistochemical expressions of FAM115C by AS were all Grade 3 in both the control group and the FAM115C-inhibited group (Fig. 4F, I). The immunohistochemical expressions of Ki-67 of the FAM115C-inhibited group were similar to those of the control group (Fig. 4G, J).

### Subcellular localization of FAM115C was observed in the cell lines and pancreas cancer specimens

**Figure 5:**
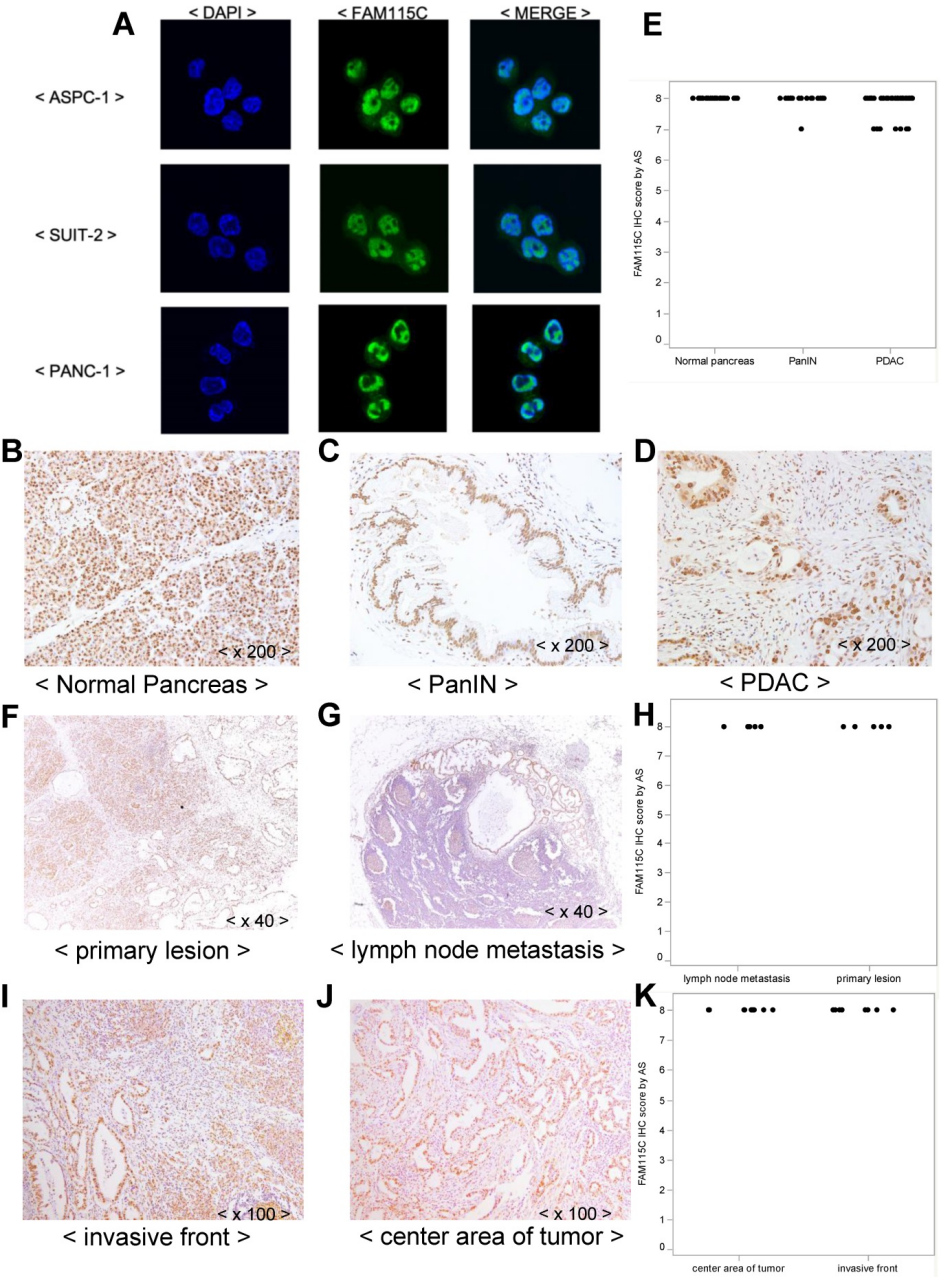
FAM115C expression patterns in PDAC cell lines. **A:** ASPC-1, SUIT-2, and PANC-1 cells were cultured *in vitro* and immunofluorescently stained for FAM115C (*green*) and nuclei (DAPI, *blue*). The localization of FAM115C expression is shown in the merged images. FAM115C was predominantly located in the nucleus although it was located in both the cytoplasm and nucleus. **B-D:** FAM115C expression patterns in normal human pancreas tissue (**B**) and resected human specimens of PanIN (**C**), and PDAC (**D**). FAM115C expression was observed ubiquitously in all these samples, and it was also located in both the cytoplasm and nucleus but mainly in the nucleus **(B-D)**. **E:** FAM115C IHC using the Allred score (AS). The AS values of the normal pancreas, PanIN, and PDAC tissue were all Grade 3. Original magnifications ×200. **F, G:** FAM115C expression patterns in primary lesions of PDAC **(F)** and lymph node metastatic lesions of PDAC **(G)** from resected human specimens. **H:** FAM115C IHC using AS. The AS values both of the primary lesions and lymph node metastatic lesions of PDAC were all Grade 3. Original magnifications x40. **I, J:** FAM115C expression patterns in invasive front lesions of PDAC **(I)** and center area lesions of PDAC **(J)** of human resected specimens. **K:** FAM115C IHC using AS. The AS values both of invasive front lesions and center area lesions of PDAC were all Grade 3. Original magnifications x100.

We examined the subcellular localization of FAM115C in the three PDAC cell lines by immunofluorescent staining. FAM115C was predominantly located in the nucleus, although it was observed in both the cytoplasm and the nucleus (Fig. 5A). We then examined the subcellular localization of FAM115C in human surgically resected specimens by IHC. In the normal pancreas tissue (acinar cells, duct cells, and islets), pancreatic intraepithelial neoplasia (PanIN) specimens, and PDAC specimens, the FAM115C expression was also located in both the cytoplasm and nucleus but mainly in the nucleus (Fig. 5B-D).

Moreover, FAM115C expression was observed ubiquitously in normal pancreas tissue such as acinar cells, duct cells and islets (Fig. 5B), in PanIN specimens (Fig. 5C), and in PDAC specimens (Fig. 5D), and all these samples were Grade 3 by AS. We thus could not evaluate the differences in FAM115C expression by IHC among the normal pancreatic tissue, PanIN specimens, and PDAC specimens.

In the case of the PDAC specimens, we could not evaluate the differences of FAM115C expression pattern by IHC between the necrotic and non-necrotic lesions of PDAC, because they were all Grade 3 by AS.

In addition, we attempted to compare the expression of FAM115C by IHC between primary lesions of PDAC (Fig. 5F) and lymph node metastatic lesions of PDAC (Fig. 5G), but we could not analyze the differences of FAM115C expression between those lesions because they were all Grade 3 by AS (Fig. 5H). In addition, we attempt to compare the expression of FAM115C by IHC between invasive front lesions of PDAC (Fig. 5I) and center area lesions of PDAC (Fig. 5J), but we could not analyze the differences of FAM115C expression between those lesions because they were all Grade 3 by AS (Fig. 5K).

### A high expression of FAM115C mRNA was correlated with better prognosis in pancreatic cancer patients

**Figure 6:**
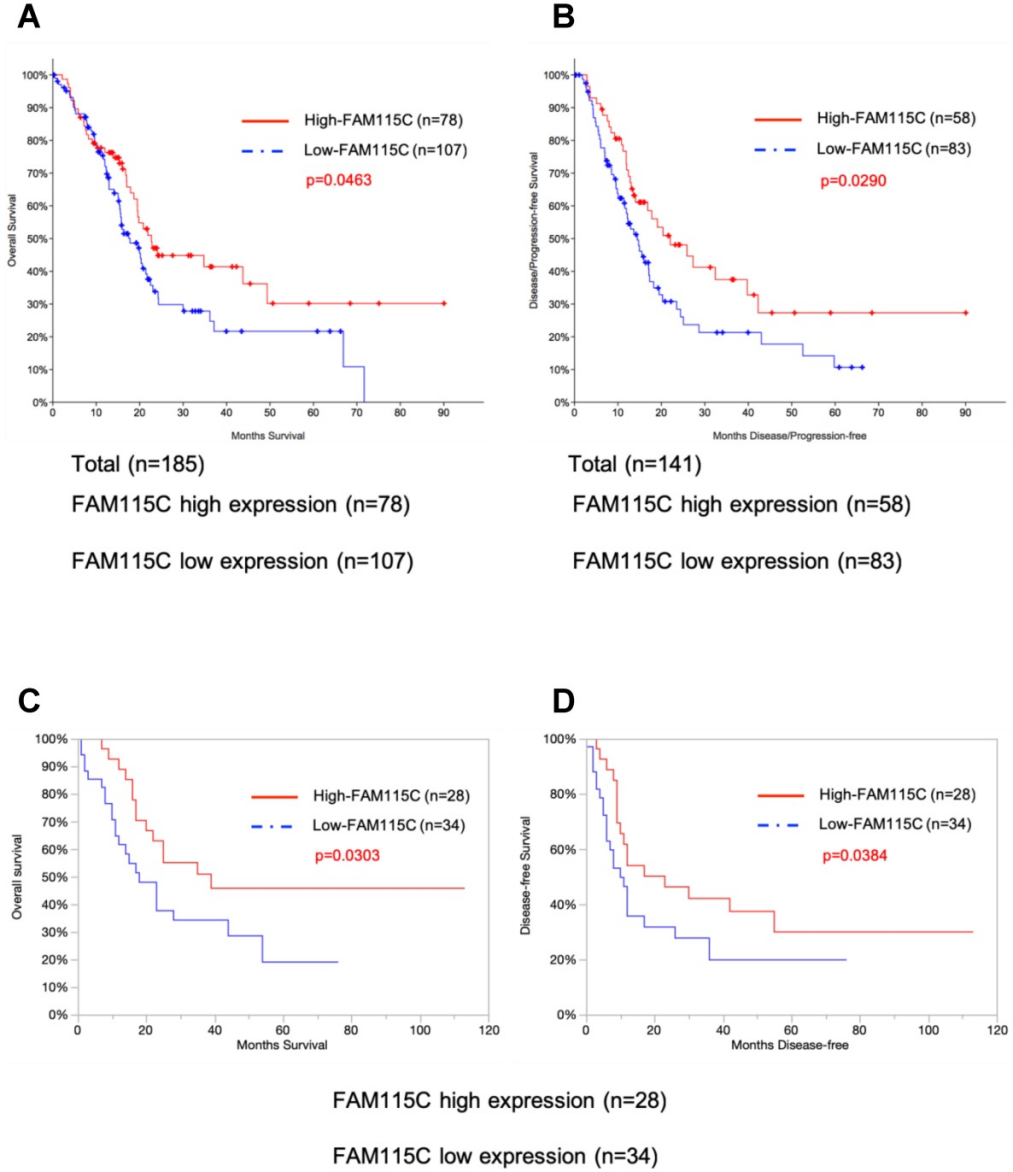
High FAM115C expression is correlated with good prognosis in PDAC patients. **A:** Kaplan-Meier analysis of overall survival using 185 PDAC patients from the publicly available TCGA-PAAD datasets (log-rank, p=0.0463). The cutoff point for the separation of the high and low FAM115C expression groups was 42%. **B:** Kaplan-Meier analysis of disease/progression-free survival using 141 PDAC patients from the publicly available TCGA-PAAD datasets (log-rank, p=0.029). The cutoff point for the separation of the high and low FAM115C expression groups was 41%. **C:** Kaplan-Meier analysis of overall survival using 62 pancreas cancer patients treated at our institution. We assessed the FAM115C expression levels of the surgically resected tumor specimens by real-time PCR (generalized Wilcoxon test, p=0.0303). The cutoff point for the separation of the high and low FAM115C expression groups was 45%. **D:** Kaplan-Meier analysis of disease-free survival using 62 pancreas cancer patients treated at our institution. We assessed the FAM115C expression levels of the surgically resected tumor specimens by real-time PCR (generalized Wilcoxon test, p=0.0384). The cutoff point for the separation of the high and low FAM115C expression groups was 45%.

We evaluated whether FAM115C mRNA expression was correlated with the survival of patients with pancreas cancer. For this purpose, we conducted a survival analysis using the publicly available TCGA-PAAD dataset (See Materials and Methods). There were significant associations between FAM115C expression and the survival periods in each of the datasets of 185 and 141 pancreas adenocarcinoma patients. Both the overall survival (p=0.0463) and the disease/progression-free survival (p=0.029) of the patients with high FAM115C expression were significantly longer than those of the patients with low FAM115C expression (Fig. 6A, B and Supplementary Tables S1,S2).

To further test the association between the FAM115C expression levels and the patients' prognoses, we enrolled 62 pancreas cancer patients treated at our institution and assessed the FAM115C expression levels of the surgically resected tumor specimens by real-time PCR. All of the patients underwent primary surgery as a standard treatment. The clinicopathological characteristics of these patients (age, sex, tumor location, tumor size, lymphatic permeation, vessel invasion, perineural invasion, tumor grade, and UICC TNM stage) are summarized in Table 1. Both the overall survival (p=0.0303) and the disease-free survival (p=0.0384) of the patients with high FAM115C expression were significantly longer than those of the patients with low FAM115C expression (Fig. 6C, D and Supplementary Tables S3, S4). Moreover, we performed a Cox proportional hazards regression analysis of the dataset of the Kyushu University cohort at 45% cutoff points, and the results showed that high FAM115C expression [HR, 0.14; 95% confidence interval (CI), 0.048-0.39; P<0.01], lymphatic permeation [HR, 2.23; 95% confidence interval (CI), 1.01-4.93; P=0.048], and vessel invasion [HR, 5.04; 95% confidence interval (CI), 1.95-13.04; P<0.01] were independent prognostic factors for overall survival (Table 3), while high FAM115C expression [HR, 0.2; 95% confidence interval (CI), 0.08-0.48; P<0.01], tumor grade [HR, 2.41; 95% confidence interval (CI), 1.14-5.11; P=0.022], and vessel invasion [HR, 2.94; 95% confidence interval (CI), 1.31-6.60; P<0.01] were independent prognostic factors for disease-free survival (Table 4). We speculate that FAM115C might have a tumor-inhibiting function in pancreas adenocarcinoma.

## Discussion

Hypoxia is a common feature in the microenvironments of solid tumors 3-7. We have shown that some notable molecules and pathways are more greatly activated under hypoxia than under normoxic conditions in PDAC cells 8,10,19,20, and we next explored new targeting molecules and pathways in PDAC cells under hypoxic conditions. In the present study's microarray analysis of PDAC cells cultured both under normoxia and hypoxia, FAM115C was highly increased under hypoxia compared to normoxia. In a further investigation, we focused on the mechanism of FAM115C in PDAC cells and investigated whether FAM115C could be a candidate prognostic marker for evaluating the outcomes of patients with PDAC.

There has been only one report about the biological mechanism of FAM115C in prostate cancer cells 11: Gkika et al. reported that FAM115C is a member of an uncharacterized protein family, which they named TCAF2 (TRPM channel-associated factor 2). FAM115C binds to the TRPM8 channel and interacts directly with TRPM8, and this promotes the trafficking of FAM115C to the cell surface. It has also been reported that FAM115C promotes migration in prostate cancer cells 11.

In our *in vitro* experiments, we observed that the knockdown of FAM115C promoted pancreatic cancer cell migration, which is the opposite of the result reported by Gkika et al. in prostate cancer. This discrepancy was likely due to the differences in cancer types between prostate cancer cells and PDAC cells.

We observed two bands in the immunoblotting of FAM115C by western blot analysis (Fig. 1B). The molecular weight of the main upper band was 101 kDa, and there was a minor lower band near the main upper band. Regarding TRPM8, the multiple-protein band was due to the glycosylation status 21. Because FAM115C is identified as a TRPM8 partner protein 11, the presence of the multiple-protein band might be due to a post-translational modification such as glycosylation. The relationship between FAM115C and glycosylation should be intensively investigated in future studies.

Our IHC results showed that FAM115C was localized to the nuclei and cytoplasm of each of the normal pancreatic acinar, PanIN, and PDAC cells and ubiquitously on consecutive sections of surgically resected PDAC specimens. We did not observe a correlation of FAM115C expression among these materials regarding the tumorigenesis.

We next investigated the correlation between FAM115C mRNA expression and the clinical prognosis in both the public database and the patients with PDAC treated at our institutions. The results of our analyses demonstrated that high FAM115C expression was associated with good prognosis in patients with PDAC. In addition to this clinical analysis, considering our finding that the inhibition of FAM115C promoted pancreatic cancer cell migration and invasion in PDAC cells *in vitro*, we speculated that FAM115C plays tumor suppressive roles in PDAC cells.

In regard to the discrepant finding that the expression of FAM115C mRNA was correlated with the prognosis of pancreatic cancer while the protein was not, this was likely attributable to differences in the expression sensitivities and evaluation methods between mRNA expression levels and protein expression levels. It has been reported that mRNA expression is more sensitive to detection than protein expression 22. And, because of the limitations of IHC for the assessment of protein expression, we could not observe a correlation between FAM115C protein expression and prognosis by analysis of the surgically resected PDAC specimens. If we could evaluate the expression levels of FAM115C protein in more detail, there is a possibility that high expression of FAM115C protein would be correlated with good prognosis.

Generally, hypoxia in tumors tends to select for a more malignant phenotype 23, increases mutation rates 24, increases the expression of genes associated with angiogenesis 25 and tumor invasion 26, and is associated with a poor prognosis 4. However, the exact molecular mechanisms and pathways underlying the effects of hypoxic conditions remain largely unknown. In this study, it is suggested that even if the pancreatic cancer is under hypoxic microenvironment, when FAM115C expression is up-regulated, FAM115C may function as a tumor suppressor and be related with patient good prognosis.

**Figure 7:**
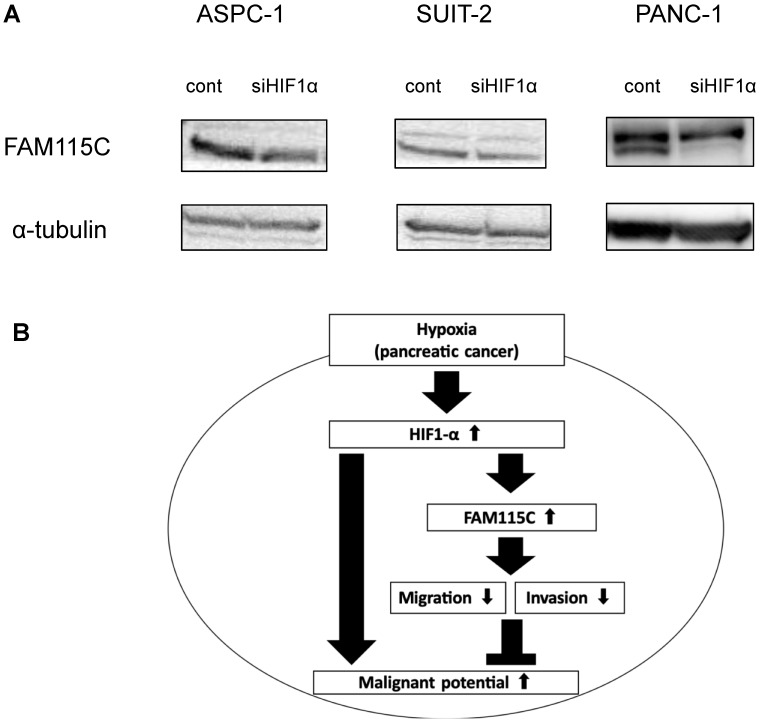
** A:** FAM115C upregulation under hypoxia might be related to HIF1α. FAM115C protein expressions in FAM115C siRNA-transfected ASPC-1, SUIT-2, and PANC-1 cells cultured under hypoxia for 24 hr were investigated by western blotting. **B:** Model of our present findings. FAM115C expression increases under hypoxia. Although it has been demonstrated that hypoxia in pancreatic cancer generally tends to proceed to malignant potential, we suggest that, based on the results obtained in this study, FAM115C under hypoxia may reduce aspects of the malignant phenotype such as migration and invasiveness in pancreatic cancer.

Hypoxia-inducible factor-1alpha (HIF-1α) was reported to be an important transcriptional factor in pancreatic cancer under a hypoxic condition 3,27. To understand the role of FAM115C in HIF1α-mediated PDAC cells, we transfected ASPC-1, SUIT-2, and PANC-1 cells with siHIF1α for 48 hr, and then cultured them under hypoxia for 24 hr. The knockdown of the expression of HIF1α in all PDAC cell lines decreased the expression of FAM115C, and especially markedly decreased in PANC-1 cells (Fig. 7A). This result suggested that HIF1α might be involved in the regulatory mechanism by which hypoxia induces FAM115C expression in PDAC cells.

In conclusion, we obtained two novel findings in the present study. First, the expression of FAM115C in PDAC cells increases under a hypoxic condition compared to a normoxic condition. Second, we showed that FAM115C could be a novel tumor suppressor in pancreas adenocarcinoma in which the microenvironment is under hypoxia, and FAM115C could be a predictive biomarker for good prognosis. Figure 7B shows the schema of our present findings. Our results may contribute to the clarification of cancer conditions and the development of new therapeutic strategies for refractory pancreatic cancer.

## Supplementary Material

Supplementary tables.

## Figures and Tables

**Table 1:** Correlations of clinicopathological characteristics between the high and low FAM115C expression groups.

Variable	High Expression (n=28)	Low Expression (n=34)	p-value
**Age, yrs ≥65/<65**	20/8	22/12	0.5985
**Male:Female**	21:7	18:16	0.1128
**Location**			
Head:Body/Tail	19:9	23:11	1.0000
**Tumor size**			
≤3.4cm	17	14	0.2016
>3.4cm	11	20	
**UICC TNS category**			
T1+2 / 3+4	2/26	3/34	1.0000
N0/ N1	11/17	9/25	0.4133
M0/ M1	28/0	32/2	0.4966
**UICC stage**			
I+IIA / IIB+III+IV	11/17	9/25	0.4133
**Tumor grade**			
Grade 1 or grade 2	11	19	0.2134
Grade 3	17	15	
**Lymphatic permeation**			
(+)	10	13	1.0000
(-)	18	21	
**Vessel invasion**			
(+)	15	20	0.7981
(-)	13	14	
**Perineural invasion**			
(+)	24	29	1.0000
(-)	4	5	

**Table 2:** FAM115C mRNA expression in chronic hypoxia-resistant ASPC-1 cells and SUIT-2 cells vs. cells under hypoxia (sild type cells) in the microarray analysis

	Z-score	Ratio
**ASPC-1**	4.976	19.7
**SUIT-2**	5.163	38.72

**Table 3:** Prognostic prediction of FAM115C by multivariate Cox regression analysis using 62 pancreatic cancer patients of overall survival at our institution.

Variable	HR (95% CI)	P value
**FAM115C**		
Low expression	Reference	
High expression	0.14 (0.048-0.39)	<0.01*
**Lymphatic permeation**		
(-)	Reference	
(+)	2.23 (1.01-4.93)	0.048*
**Vessel invasion**		
(-)	Reference	
(+)	5.04 (1.95-13.04)	<0.01*

*Significant difference

**Table 4:** Prognostic prediction of FAM115C by multivariate Cox regression analysis using 62 pancreatic cancer patients of disease-free survival at our institution.

Variable	HR (95% CI)	P value
**FAM115C**		
Low expression	Reference	
High expression	0.2 (0.08-0.48)	<0.01*
**Tumor grade**		
Grade 1 or grade 2	Reference	
Grade 3	2.41 (1.14-5.11)	0.022*
**Vessel invasion**		
(-)	Reference	
(+)	2.94 (1.31-6.60)	<0.01*

*Significant difference

## Abbreviations

AS: Allred score
FAM115C: human family with sequence similarity 115, member C
IHC: immunohistochemistry
NCBI: National Center for Biotechnology Information
PanIN: pancreatic intraepithelial neoplasia
PDAC: pancreatic ductal adenocarcinoma
TCGA-PAAD: The Cancer Genome Atlas-Pancreatic Adenocarcinoma
TRPM8: transient receptor potential 8
