# Supplementary material for: FAM115C could be a novel tumor suppressor associated with prolonged survival in pancreatic cancer patients
Source: J Cancer. 2020 Feb 10;11(8):2289–302. doi: 10.7150/jca.38399 (PMC7052938; doi:10.7150/jca.38399)
Supplement: Supplementary file 1 — Supplementary tables. [file jcav11p2289s1.pdf]

Supplementary Table S1.

Prognostic capability of FAM115C on the overall survival of 185 PDAC patients from the publicly available TCGA-PAAD dataset.

variable	P value
20%	0.075
25%	0.148
30%	0.321
35%	0.139
40%	0.146
42%	0.0463*
45%	0.208
50%	0.186
55%	0.274
60%	0.261
65%	0.507
70%	0.781
75%	0.959
80%	0.520

*Significant difference

At the cutoff point of 42% for the separation of the high and low FAM115C expression group, high FAM115C expression is correlated with good prognosis of overall survival. P value; log-rank test.

Supplementary Table S2.

Prognostic capability of FAM115C on the disease/progression-free survival of 141 PDAC patients from the publicly available TCGA-PAAD dataset.

variable	P value
20%	0.274
25%	0.156
30%	0.196
35%	0.033*
40%	0.039*
41%	0.029*
45%	0.207
50%	0.398
55%	0.222
60%	0.497
65%	0.986
70%	0.853
75%	0.934
80%	0.443

*Significant difference

At the cutoff point of 41% for the separation of the high and low FAM115C expression group, high FAM115C expression is correlated with good prognosis of disease/progression-free survival. P value; log-rank test.

Supplementary Table S3.

Prognostic capability of FAM115C on the overall survival of 62 pancreatic cancer patients treated at our institution.

variable	P value
20%	0.1898
25%	0.1115
30%	0.0799
35%	0.2094
40%	0.0954
45%	0.0303*
50%	0.1894
55%	0.1094
60%	0.2089
65%	0.3357
70%	0.0741
75%	0.0461*
80%	0.1574

*Significant difference

At the cutoff point of 45% for the separation of the high and low FAM115C expression group, high FAM115C expression is correlated with good prognosis of overall survival. P value; generalized Wilcoxon test.

Supplementary Table S4.

Prognostic capability of FAM115C on the disease-free survival of 62 pancreatic cancer patients treated at our institution.

variable	P value
20%	0.6693
25%	0.2766
30%	0.1623
35%	0.3507
40%	0.1547
45%	0.0384*
50%	0.2106
55%	0.1707
60%	0.3516
65%	0.6277
70%	0.2103
75%	0.1922
80%	0.0363*

*Significant difference

At the cutoff point of 45% for the separation of the high and low FAM115C expression group, high FAM115C expression is correlated with good prognosis of disease-free survival. P value; generalized Wilcoxon test.
